# How to provide existential and spiritual support to people with mild to moderate dementia and their loved ones. A pilot study

**DOI:** 10.1371/journal.pone.0298783

**Published:** 2024-03-27

**Authors:** Marc Haufe, Saskia Teunissen, Carlo Leget

**Affiliations:** 1 Department of Care Ethics, University of Humanistic Studies, Utrecht, Utrecht, The Netherlands; 2 Julius Center for Healthcare Sciences and Primary Care, University Medical Center Utrecht, Utrecht, The Netherlands; University of KwaZulu-Natal College of Health Sciences, SOUTH AFRICA

## Abstract

**Background:**

People with mild to moderate dementia and their loved ones may experience strong existential and spiritual challenges due to the disease. People with dementia could therefore benefit greatly from ongoing conversational support. Within the literature and in supportive practice, there are very few tools that help professionals provide this type of support. Professionals may therefore be unaware of, or uncertain of, how support can be given.

**Objective:**

To develop and test support approaches that may enable professionals to better conduct conversations with attention for existential and spiritual issues.

**Methods:**

Participatory action research was conducted with dementia care professionals who spoke to 62 clients and 36 loved ones. Research consisted of two cycles of analyzing support, formulating strategies to try, testing and reflecting on the success of these actions and formulating new ones. The Diamond model for existential and spiritual issues regarding mild to moderate dementia, developed in previous research, was used as a framework.

**Results:**

Five types of approaches, corresponding to the five fundamental polarities within the basic framework, were found to be helpful in alleviating tensions and bolstering strengths. For issues of self-confidence and -worth, an approach of *exploring the felt self* was developed; for issues of capacity and adaptability, an *exploring daily routines* approach; for issues of security and loss, an *exploring a trinity of needs* approach; for issues of burden and enrichment, an *exploring memory* approach; and for issues of faith and meaning, an *exploring ones’ predicament* approach. When exploring these approaches, participants found sets and sequencing of questions and prompts to be helpful and transformative.

**Conclusion:**

Professionals can use the Diamond framework to provide conversational support to alleviate tension, enhance meaning and bolster strength for clients and loved ones.

## Introduction

More than 55 million people live with dementia globally, with approximately 10 million new cases each year. Dementia is a dominant cause of disability among older people [1]. People with mild to moderate dementia and their loved ones can grapple with a host of existential and spiritual issues when dealing with the impact of the disease [2–4]. Especially in these stages, where sense of self, agency and social connections are affected, people may struggle to find meaning and purpose in life [5, 6]. People may find that they need support that enables them to get more in touch with themselves and what matters most to them [7]. Setbacks experienced due to cognitive decline occur regularly and may trigger a new search for meaning and inner peace [2, 8, 9]. In these stages therefore, people and their loved ones may benefit greatly from proactive care with regular follow-ups to help them with existential and spiritual issues [6, 10, 11].

Within the literature, and in practice, there is a lack of substantiated models and methods that would enable existential and spiritual care specifically for people with mild to moderate dementia and their loved ones [4, 11, 12]. Dementia care professionals can therefore experience uncertainty and lack of confidence in trying to attend to existential or spiritual needs [13–15]. Healthcare profesionals need to better understand existential and spiritual issues of people with mild to moderate dementia and ways to explore these issues [15–17].

**Fig 1:**
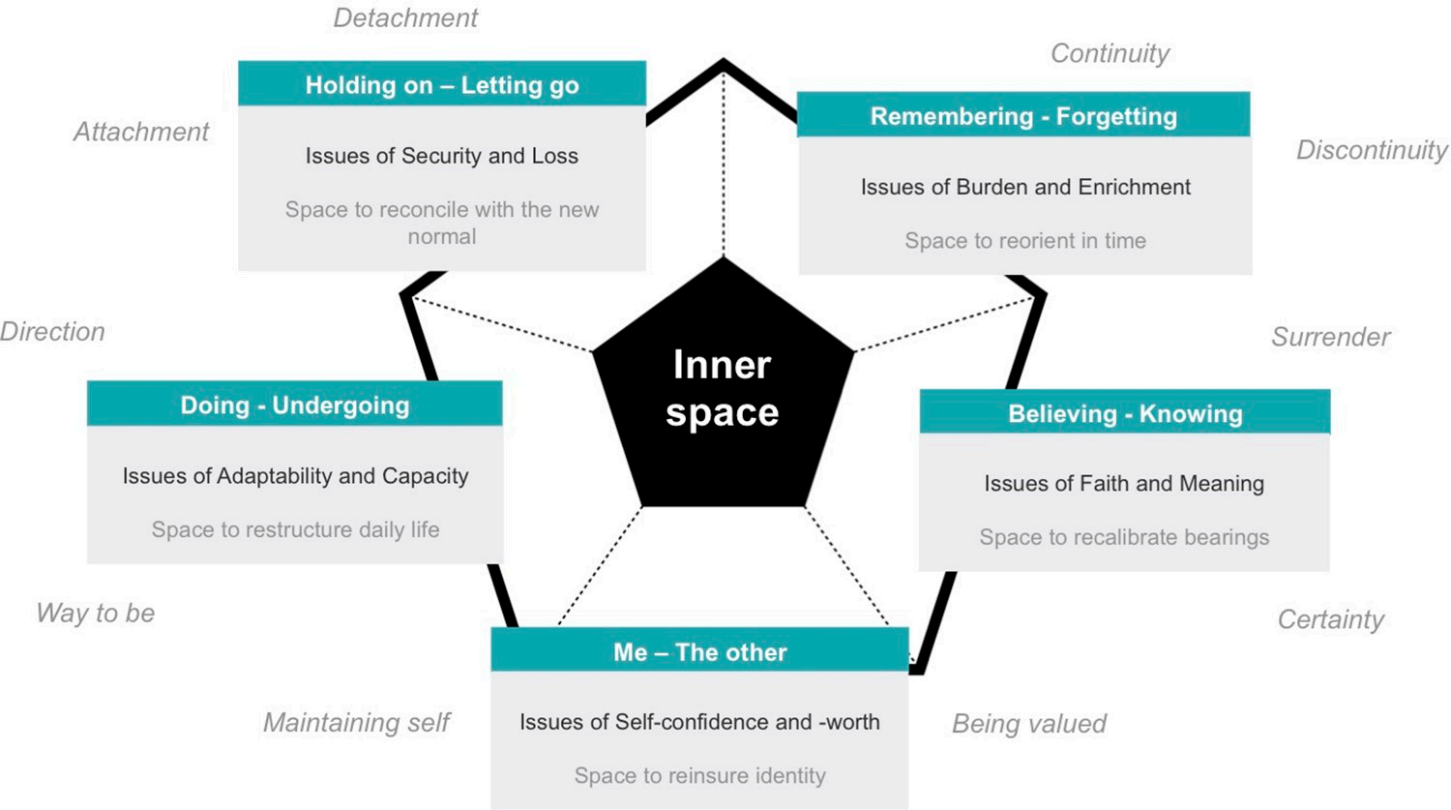
The Diamond spiritual conversation model for mild to moderate dementia.

In previous research we have developed a conversation model that can facilitate ongoing existential and spiritual support for people with mild to moderate dementia and their loved ones [18]. The model, see Fig 1, is based on a broadly used and vailidated spiritual conversation model in Dutch and Belgian palliative care settings [19–22].

The model comprises five existential or spiritual polarities that help to discuss important issues that can arise. The *me–other* polarity is about changes in the way a person relates to self and the way others affect that relationship. The *doing–undergoing* polarity concerns changes in the ability for a course of action. The *holding on–letting go* polarity deals with changes in important relationships. The *remembering–forgetting* polarity concerns changes in how someone relates to memories. *The believing—knowing* polarity is about changes in the way one relates to things greater than oneself. For early stage dementia, specific issues and accents were found to be prevalent within these polarities [18]. The Central idea is that fostering inner space is important to cope with existential or spiritual tension, and to finding strength. Inner space is understood as an experience of open receptiveness, wherein issues can be overseen reviewed and related to with a sense of freedom [18, 19].

The model can help healthcare professionals, clients and loved ones become more aware of important existential and spiritual issues. However, it has not yet been applied as a tool to help shape conversational support. Such tools are greatly needed in the field [11]. In this research we have therefore applied, and further developed, the model in practice to incorporate specific types of exploratory approaches that help enhance the experience of inner space. We developed a set of tested practical approaches and questions within the model’s framework that can help caregivers better support clients and their loved ones.

## Materials and methods

### Research paradigm and qualitative approach

We conducted participatory action research with dementia care professionals to develop specific exploratory approaches regarding the polarities of the Diamond model. In participatory action research, researchers and participants conduct self-reflective inquiry to better understand and improve the practice in which they participate [23]. Researchers and practitioners analyze the practice together, formulate ideas on how to improve, implement the ideas and evaluate the changes each from their own perspective. In doing so, scientific and practical knowledge can inform each other and become more integrated, making that knowledge more grounded in practice and more actionable for professionals [24]. This approach suited our research purposes well as we had the double goal of further grounding our scientific model for spiritual conversations in practice, and developing that model to include more practical ways to conduct conversations. Our research consisted of four phases.

### Phase one: Agreeing upon action research goals

In the first phase (September 2021), the Diamond model for early stage dementia was presented by the researcher to five case managers and one psychologist who had previously indicated their willingness to participate in action research with the goal of improving existential and spiritual support. In the Netherlands, dementia case managers are specially educated nurses or social workers on a university of applied science level. They provide ongoing emotional and practical support for people with dementia who are still living at home and their loved ones. They are trained in making people aware of, and helping them secure, social and care services, providing information about the disease and providing socio-emotional supportive conversations. The case-managers were highly motivated to reflect on their ongoing conversations with clients and loved ones, with the help of the model, to develop more successful ways of exploring existential or spiritual issues. The psychologist was tasked with designing, and further developing, peer discussion groups with clients using the Diamond model as a guiding framework. She too wished to develop approaches to explore existential and spiritual issues in such a way that people with dementia felt more support. All professionals agreed to develop their approaches using the model as the framework. To that end they were given a picture of the model to carry with them to help them analyze and shape their conversations.

### Phase two: Relating the Diamond model to practice

In the second phase (October 2021) professionals became more fluent with the model by using it as an awareness enhancing and generative tool. At the end of the month, one-on-one reflective co-constructive interviews between the principle researcher and the professionals were held. One-on-one interviews were chosen because they allowed for more in depth reflection on individual practices and provided safe spaces for participants to open up about experienced difficulties. It also allowed for maximum flexibility in accommodating the participants’ very busy schedule. Due to restrictions of personal contact following the onset of the corona virus, all research activities were conducted in an online environment. Phase two interviews focused on analyzing ongoing cases with the help of the model, and reflecting on exploratory approaches that had helped, or might help, in enhancing the experience of inner space. In this phase at the end of supportive conversations, clients and loved ones were more regularly asked if they felt less burdened or more energized as a result of the (group) conversation, as a self-reported measure of inner space enhancement.

### Phase three: Testing phase two learning’s and incorporating them into the model

In phase three (November 2021), professionals continued to use the model as an awareness and generative tool for conversation support on existential and spiritual issues. End of phase interviews again consisted of analyzing ongoing cases and reflecting on exploratory approaches that enhanced the experience of inner space. Professionals reported that they had become more aware of how existential and spiritual issues could arise and play out in their conversation with clients and their loved ones. Based on this awareness, they were able to engage more consciously in existential and spiritual exploration and support. At the end of this phase successful approaches for exploration and the enhancement of inner space was more clearly reported by the professionals than in phase two.

### Phase four: Member checking the found exploratory approaches

In phase four (February 2022) the researcher coded the transcripts of the phase three and four interviews, identifying the most helpful exploratory approaches and questions for each of the five polarities of the model. These approaches and questions were then collected and sent via email to the participating professionals to check for accuracy. Following this member check, final adjustments in the wording of the approaches were made.

### Researcher characteristics and reflexivity

The main researcher conducted this study as part of his PhD research into spiritual support for people with mild to moderate dementia and their loved ones. The researcher became aware over the course of his PhD research that such support was still very much an open field for researchers and dementia caregivers alike. At the same time, the researcher was confronted with an ongoing struggle to help his father living with moderate dementia (when conducting this research) with his existential and spiritual issues. Though trained as a social psychologist and having extensive experience with qualitative techniques, personal experiences showed the researcher clearly that this prior knowledge was inadequate. This research thus not only presented an opportunity to gain more scientific insight into the dementia field, but also to learn from the clinical experience of professional dementia caregivers. This dual pursuit has had an influence on the choice for a participatory action research design. In this design researcher and co-researching practitioners engage with one another in interpersonal reflexivity. In reflecting on the struggles within current supportive practice and co-constructing new ways to aid clients existentially and spiritually, the researchers own experiences have also been shared and utilized. On the one hand this type of positioning has made the researcher less of an impartial sounding board, on the other hand this positioning has encouraged openness about the vulnerability of being a caregiver and a sense of equality between researcher and participating professionals.

### Research context

Research was done with the participation of a dementia care organization that offers support to people still living at home. Through the organization people with early stage dementia can receive ongoing support from case managers who help people and their loved ones deal with the impact of the disease on daily life. Case managers conduct regular at home conversations with clients and loved ones where they explore any difficulties they may be experiencing and possible solutions. Within these conversations, existential and spiritual themes may be prevalent, but case managers are generally not specifically trained to recognize or explore these themes. This research was seen as a good way to bolster that ability. Clients are also offered opportunities by the organization to participate in peer discussion groups held on the premises of the dementia care organization. These discussion groups, facilitated by a psychologist, enable clients to express and exchange experiences, so that they can learn from and support one another. Within the organization, a new type of discussion group for people with early onset dementia (roughly for people between 50 en 68 years of age) was being developed. The psychologist tasked with this development was searching for a new way to approach discussions groups. Our research offered a way to do this.

### Sampling strategy

A mixed strategy of convenience and purposeful sampling was used. Through our previous research, where we developed the general framework of the Diamond model for mild to moderate dementia, we had already established a good working relationship with key personnel (a chaplain specialized in dementia care and the manager of the case managers) in the aforementioned dementia care organization. We were able to recruit participants through them. It was estimated that the case managers and the psychologist would see a bigger diversity of primary dementia forms (Alzheimer, Vascular, Frontal lobe) and generate a fairly equal distribution across age and gender in the allotted research time frame.

### Ethical issues pertaining to human subject

The University Medical Center of Utrecht ethics review board evaluated the study. They established that the Dutch Medical Research Involving Human Subject Act (WMO, art.1b) did not apply, as offered support was not deemed medically invasive. Approval of this study by the review board was therefore not required (WAG/mb/18/038341). Consent for participation of the professionals was obtained in written form via email one week after the goals of the research, it’s participatory and confidentiality principles and data handling procedure had been explained to them in an exploratory opening session. All information about the existential and spiritual issues and processes of clients and their loved ones was handled under the provision of anonymity. The main focus of the research was to collect successful exploratory forms, not to probe client’s specific experiences.

### Data collection, processing and analysis

Data collection started on 01-09-2021 and ended on 25-02-2022. Types of data included interview recordings and transcripts, researcher notes and notes of the participating dementia care professionals. All data were stored on a secured network at the University of Humanistic studies under the provision of anonymity. Clients and loved ones were not named in the interviews and notes and results shared were only reported on an aggregate level. The reflective interviews of phase two and three, were all structured with the help of a loose guideline. The guideline questions were:

- Which existential or spiritual issues did you encounter in the previous weeks?
- Which approaches and or questions did you use to explore these issues?
- Which approaches contributed to the enhancement of inner space for clients and or loved ones?

Interviews lasted between 45 and 90 minutes, with a mean of 60 minutes. All interviews were recorded with Microsoft Teams and transcribed by an agency using a secured connection. The conversational approaches and questions, which were reported by the participating professionals to lead to more inner space with clients and loved ones were coded and extracted from the transcripts by the principal researcher with the help of Atlas Ti 9. Approaches and questions were shared with the other authors, discussed and divided in approaches to explore tension or to explore strength, as these two exploration pathways were seen to be dominant. General principles for the enhancement of inner space were also discussed and formulated. Trustworthiness of data was achieved through having multiple disciplines reflect on, and test, how Diamond model issues could be explored, and by member checking. Types of approaches and questions that participants felt they had used successfully repeatedly were selected for final reporting.

### Participants

In total, 5 case managers and 1 psychologist took part, supporting 62 clients with the help of the Diamond model. The participating professionals were a fairly mixed group in terms of age, years of experience with the client group. The mean age of the professionals was 42,8 years, with the youngest being 34 years of age and the oldest 56. Regarding years of experience with the client group the mean was 7,5 years, whereby the professional with the most experience had 22 years experience and the professionals with the least experience 1 year.

The clients supported with the help of the Diamond model consisted of two groups. One older group (mean 83 years old, youngest 54 years, oldest 66 years) of patients who were supported at home by the case managers and one younger group of people with early onset dementia (mean 61 years old, youngest 71 years, oldest 96 years) who lived at home, but came to the healthcare facility for discussion groups. Concerning the former most supportive conversations were conducted with a loved one present (for 36 of 46 clients). Clients were more female (n = 45) than male (n = 17). In total 62 clients were supported. All were scored mild, or mild to moderate in the Clinical Dementia Rating [25]. Most clients had Alzheimer’s disease (n =, 34) or a combination of Alzheimer’s and Vascular dementia (n = 9), but a fair distribution among other types of dementia particularly vascular (n = 10) and to a lesser extend Frontotemporal (n = 5) and Parkinson dementia (n = 4) was present.

## Findings

Over the course of our participatory action research different approaches for working with the diamond model for early stage dementia have been identified.

Below we first present a general process regarding the nature of existential or spiritual conversational support that has as its primary goal the enhancement of inner space. This is followed by a specification of how these insights pertain to five polarities of the Diamond model.

### General process for the enhancement of inner space

#### Tuning into the existential or spiritual

Not all aspects of the conversations that professionals had with people with mild to moderate dementia and their loved ones were seen as existentially or spiritually relevant. There were conversational cues that could activate a more existential or spiritual approach. Or as one case manager put it:

*“It is because of such signals that I feel the necessity to go underneath the surface of things…”* (Case manager 2, interview 2)

Such signals, mostly apparent trough facial expression and body language, could take on a variety of forms but can roughly be classified as signals of *tension* due to experienced difficulties and challenges, and signals of *strength* and a need for growth, as the two quotes below illustrate.

*“For me a signal is definitely that I see someone struggling and I may ask*: *‘I am picking up signals that this is very difficult for you*, *is that correct*?*’”* (Psychologist, interview 1)

*“Sometimes I can notice*: *‘ok this lady needs much more space*, *she needs space from me to grow within the context of our conversations…’”* (Case manager 3, interview 2)

#### Turning into a co-researcher

Many respondents reported that following up on such signals required an inner movement or repositioning on their part to open up a space for the existential or spiritual dimension in the conversation. This was required because much conversation tended to follow a certain protocol that is not conducive to an open exploration of the deeper concerns of clients and loved ones:

“We have standard questionnaires that we often use in conversations, but sometimes you notice that you really need to disregard those questions and just open up to what people want to say” (Case manager 3, interview 1)

*“You need to create space for that and that is a decision point for me*, *a decision to make space in myself to be able to receive”* (Case manager 5, interview 1)

This inner movement was also described metaphorically as turning from standing opposite someone to standing next to someone in the conversation:

*“You have to go and stand next to someone*, *in that moment you are not there to teach or advise*, *but to be a kind of co-researcher…”* (Psychologist, interview 2*)*

Standing next to each other as co-researcher was reported to open up a shared view to (part of) someone’s life experience.

#### Exploring experiences: The Diamond model polarities

Once in this mode of co-researching, the existential or spiritual issues could better be explored. A dominant way to start doing this concerned asking for concrete experiences as examples of how the issues manifest themselves in peoples lives.

*“I always ask for examples*… *that way you really can spotlight a moment*… *it’s like watching a little movie together”* (Psychologist, interview 2*)*

Such prompting was found to often stimulate an unfolding story to listen to or research together in terms of existential or spiritual tensions or strengths. These stories could be listened to and further explored with the help of the Diamond model. In this way issues, themes and storylines could be related to the Diamond polarities, prompting specific conversational approaches. These results will be taken up in the next section.

#### Pregnant silences and key transformative questions

After a thorough exploration together of existential or spiritual tension or strength, there was said to often be a moment of pregnant silence. This was found to be an especially important moment following the exploration of tensions.

*“When the tension is fully named*, *then they can be silent”* (Case manager 4, interview 2)

*“There often follows a moment of silence*, *where people are processing what has been said*. *It is important to give that time*, *that there is space for that*.” (Psychologist, interview 2)

Following such silent contemplation it was found that certain key questions can be asked to facilitate a calling forth of a core perspective on the matters at hand.

*“…and from there you can proceed with*: *‘what is most important*?*’*” (Psychologist, interview 2)

*“What does it mean*?*”* (Case manager 1, interview 2)

*“What is needed*?*”* (Case manager 4, interview 2*)*

#### Room for possible alternatives and strengths

Answering these questions was found to alleviate the tension from conflicting thoughts and feelings and freedom and space to move forward.

*“Its like the tension subsides making room for the exploration of possible alternatives*.*”* (Psychologist, interview 2)

In this sense, even though professionals explored both tensions and strengths together with people, the experienced tensions were often leading in the conversations. It was found that trying to explore strengths or new avenues of potential could often not be done without getting to the core meanings or needs of tensions first. Often, professionals would therefore explore the tensions first and then seek to transition to the exploration of strengths. This could be done in one conversation, but also across a number of conversations. As mentioned above, both types of exploration could be aided by the use of the Diamond model polarities, to which we now turn.

#### The Diamond model polarities: Exploring experiences

Below we present what we found to be successful exploratory approaches for each of the five polarities within the Diamond model. In the phase two and three interview, professionals reflected on conversational strategies that they wanted to use, or had used, in order to become more aware of the value of those approaches. Such reflection provided not only new insight into the many benefits of the enhancement of inner space but also new approaches and transition questions to uncover existential or spiritual tensions or strengths.

#### Me–Other: Exploring the felt self

This polarity deals with issues of self-confidence and worth, as they relate to the experience of *maintaining a sense of self* or *being valued* by others. It was found in our research that felt changes in intensity of emotions and fulfillment of important roles often led to felt tensions and a need for support. This type of support was given most in two peer group discussions with people with early onset dementia, which specifically dealt with issues of staying connected to a sense of self. A key opening question in these groups was:

“Do you feel yourself?” (Psychologist, interview 1)

#### Tensions regarding the felt self

Answers to this question were frequently negative, with people commenting on feeling some sort of tension regarding loss of self. Listening to the unfolding stories with the help of the polarity helped to hear tensions regarding maintaining a sense of self with an intrapersonal aspect or an interpersonal aspect. Intrapersonal tension often dealt with feeling out of place within oneself due to the effects of the disease. This tension could be further explored by asking questions about the accompanying thoughts, mood/ emotions and the body with questions such as:

*“What kind of effect does it have on your mood*?*”* (Psychologist, interview 1)

*“What are dominant emotions that come up*?*”* (Case manager 4, interview 2)

*“What kind of effects does it have on your bodily experience*? *For instance does it affect your breathing or energy level*?*”* (Psychologist, interview 2)

Interpersonal tension often dealt with important roles for people, such as father, provider or wife and the ways in which people felt they could not fulfill such role anymore. This tension could be further explored by asking about important relationships and the way other people react differently to the person with dementia.

*“*How do you relate to others that are important to you?*”* (Psychologist, interview 1)

*“What are noticeable changes*?*”* (Psychologist, interview 2)

*“How do they react*?*”* (Case manager 1, interview 1)

#### Key transformative questions

After exploring these identity related tensions helpful key questions were found to be:

*“What do you need to feel yourself*?*”* (Psychologist, interview 1)

*“What do you need from others to feel yourself*?*”* (Psychologist, interview 2)

Answering these questions could give the client a new perspective and free up some inner space. This, in turn, could also allow for a transition to exploration of possible strengths.

#### Strengths regarding the felt self

Asking questions about types of situations, activities and people that made people feel strong helped identify aspects of the felt self that needed to be heeded or nurtured.

*“Because sometimes people can sort of forget about those instances*, *and it can be very valuable to get in touch with that or relive that so it can guide them*.*”* (Psychologist, interview 2)

For being valued, special attention was paid to the exploration of all the ways important roles are fulfilled. This was successfully prompted by the questions:

*“What are various ways you fulfill your role as [father*, *wife etc*.*]*?*”* (Psychologist, interview 1)

*“How are you of value in that role*?*”* (Psychologist, interview 1)

*“How can you be of value in that role*?*”* (Psychologist, interview 2)

An extra boost for experiencing both types of strengths was found to be explorations of character traits, qualities or talents that were regarded as positive by self or others.

*“*…*then you really see them grow*!*”* (Psychologist, interview 2)

#### Doing–Undergoing: Exploring daily routines

This polarity concerns issues of adaptability and capacity as they relate to a sense of *direction* in what to do and *a way to be* in daily life. Dementia was often found to cause a variety of challenges, such as overstimulation or loss of initiative, which made going about ones day-to-day business difficult. This polarity came up in both the group discussions held by the psychologist and the support conversations conducted by the case-managers. An important question to open up exploration was:

*“How are you managing your daily life*?*”* (Psychologist, interview 2)

#### Tensions regarding daily routines

For both people with dementia and their loved ones managing the daily routine could cause tensions. Regarding a sense of direction, tensions were often experienced between the person with dementia and others. Others where experienced as interfering with the way the client wanted to conduct his daily life. Loved ones for instance, could direct the person with dementia to keep doing things, such as certain social activities, that the person did not want to do anymore. Case-managers too, could be perceived as someone who was there to tell the person what he could or could not do. Clients could offer a lot of resistance to this perceived interference, especially if they did not recognize or agree with the diagnosis.

*“In those circumstances you can’t really offer advice*, *because the person will not want to take it*.*”* (Case manager 3, interview 2)

A path forward was a further exploration of this tension with the help of questions like:

“*How are people [am I] interfering*?*”* (Case manager 2, interview 1)

*“How does that impact you*?*”* (Psychologist, interview 1)

Regarding the pole of a way to be, people with dementia could also feel tension in not being able to carry out certain activities, such as driving a car or doing the grocery shopping. Losing the ability to plan and organize or maintain an overview, were often seen as an impediment on daily activities. Questions used for further exploring this type of tension were:

*“What are the specific challenges that [the activity] represent*?*”* (Case manager 3, interview 1)*)*

*“How does that differ from before*?*”* (Case manager 3, interview 1)

#### Key transformative questions

Concerning these challenges of daily life, helpful key contemplative questions, also with regard to a transitioning to strengths, were found to be:

*“What is most important for you going forward*?*”* (Case manager 3, interview 2) *“What does [this activity or pastime] mean to you*?*”* (Psychologist, interview 2)

#### Strengths regarding daily routines

Regarding strengths, two types of explorations were found to be helpful. Concerning direction, the case-managers especially, explored areas where people with dementia had or could have a sense of choice and control. Regarding their own role as a possible interfering agent, an open question giving maximum control to the person with dementia was:

*“What would you like to talk about*?*”* (Case manager 3, interview 2)

Exploration of wishes for the future was also found to offer a sense of direction based on what was most important to people, with questions such as:

*“How do you see the future*? *What do you want to keep doing for as long as possible*? (Case manager 4, interview 1)

*“What would be ideal living conditions if staying in the current home would not be possible any longer*?*”* (Case manager 3, interview 2)

Concerning strengthening a way to be, exploration of valuable or pleasurable activities or pastimes was found to be supportive, particularly in the discussion groups. Valuable questions were:

*“What [pastimes or] activities do really enjoy*?*”* (Psychologist, interview 2)

*“Which elements of this pastime or activity are particularly pleasurable or relaxing*?*”* (Psychologist, interview 2)

A special focus in this exploration was also a search for analogous activities or pastimes that had the same type of meaning or experiential elements as those that were not possible any longer.

#### Holding on–Letting go: Exploring a trinity of needs

This polarity concerns issues of security and loss as they relate to *attachment* to what is familiar and *detachment* from aspects of that familiarity. The relationship between the person with dementia and important attachment figures, mostly the partner, was often found to play a central role. Partners were found to be compatible or incompatible in their attempts to come to grips with the constantly changing situation. For instance, a person with dementia could let go of the more certain aspects of his former life, whilst the loved one might be holding on tightly to the ways things were. Challenges of attachment and detachment frequently came up in supportive conversations of the case managers, often conducted with both the person with dementia and their partners, but also came up in discussions conducted by the psychologist. A good opening question was found to be:

*“How are you both coping with experienced changes*?*”* (Case manager 3, interview 1)

For the professionals, exploration of this polarity often had three sides: the person with dementia, the loved one and the relationship itself.

#### Tensions regarding a trinity of needs

Responses to this question could reveal difference between the person and their loved one either verbally or non-verbally.

*“There were instances when the client said one thing and the partner signaled that they had a very different view*.*”* (Case manager 5, interview 1)

For exploring this tension further, case-managers often conducted a double line of inquiry. For the attachment pole, where person and loved one had a strong sense of commitment to one another, such separate lines of inquiry were often conducted in the same conversation, whereby the experienced differences could be mirrored. Important differences were found in the way dementia related changes were viewed, the types of concerns regarding these changes and the type of expectations partners had about each other. Questions conducive to this mirroring process were:

*“Do you experience that as well*? *How does it differ*?*”* (Case manager 3, interview 1)

*“How do you experience hearing this from [the other]*?*”* (Case manager 3, interview 1)

For the detachment pole, where person and loved one could not really relate to each other, this type of exploration was not found to be conducive. Here, conversations apart from each other were more beneficial in exploring experienced tension with questions like:

*“Do you feel understood by your partner*?*”* (Case manager 1, interview 1)

*“What does what no longer is possible together mean to you*?*”* (Psychologist, interview 1)

*“Do you have trouble moving on*?*”* (Case manager 3, interview 2)

#### Key transformative questions

For gaining a core perspective and a (relative) sense of release from tension within this polarity, key questions were said to be:

*“What do you need from each other/ the other*?*”* (Case manager 1, interview 1)

*“What do you need for yourself*?*”* (Case manager 3, interview 1)

#### Strengths regarding a trinity of needs

To get in touch with (potential) strengths, differing ways of exploration were helpful. Regarding attachment, an exploration of reciprocity, mutuality and shared enjoyments could help people get in touch with the strengths of their relationship. Facilitating here were questions like:

*“How are you complementary to each other*?*”* (Case manager 3, interview 1

*“What do you both enjoy*?*”* (Psychologist, interview 1)

With respect to detachment, conversational support regularly transitioned to individual felt self or daily life explorations in order to strengthen the person with dementia or the loved one in his or her own life.

*“Sometimes people just need a time-out for their own story and needs”* (Case manager 3, interview 2)

Often, case managers would then also make an offer of additional conversational support in the form of peer discussion groups. These types of time outs from the ongoing pressures could then offer a chance to deal with a sense of loss and reposition one self in the relationship.

#### Remembering–Forgetting: exploring memory

With respect to this polarity, issues of burden or enrichment of memory come up as people relate to a *continuity* of the more distant or a *discontinuity* of the recent past. With Alzheimer’s disease, but also with other forms of dementia, changes in the functioning of memory were repeatedly found to cause challenges for the person and or the loved one. This could concern intense onset of early memories or forgetfulness. Both case managers and the psychologist often dealt with this polarity. A good starting question was found to be:

*“How are you experiencing memories or remembering*?*”* (Case manager 3, interview 1)

#### Tensions regarding memory

Tension filled stories and examples often dealt with challenges concerning the resurgence of distant memories or forgetfulness, the latter especially for people with Alzheimer’s. Continuity of distant memories could be especially troublesome if they dealt with unresolved conflict or trauma. A fuller exploration of such memories was done by traveling back in time with the person with the help of questions concerning emotion and relationships:

*“What are dominant emotions that go with the memory*?*”* (Case manager 4, interview 1)

*“What was the role of others*?*”* (Case manager 4, interview 1)

Sometimes, though, the complexity of the conflict or intensity of the trauma could be too great for general ongoing support conversations. Professionals would then refer their clients to specialized trauma care.

Grappling with forgetfulness could not only cause tension for the person with dementia, but also tension between person and loved one. More exploration of this was done with the help of a questions like:

*“When is it most a hindrance [to either of you]*?*”* (Case manager 3, interview 1)

*“How does it keep [either of] you from attending to the things you want to do*?*” (*(Psychologist, interview 2)

Some case managers also remarked that they too could experience a level of tension in supporting people with this pole, especially *across* supportive conversations:

*“…and it can become rather unpleasant if you have to repeat such explorations… you could say that it interferes with my ability to make space for this issue*.*”* (Case manager 4, interview 1)

This repetitiveness of exploration could also be a factor in exploring the other polarities.

In order to keep making space repeatedly, one case manager found it helpful to call to mind a specific perspective:

*“People with whom the person has daily contact experience this much more frequently… In this moment [this person] can again get to a sense of release*. *This is how I can support [them*].” (Case manager 4, interview 1)

#### Key transformative questions

For the enhancing of inner space on the above issues, facilitating questions were found to be:

*“What does this memory mean to you*?*”* (Psychologist, interview 2)

*“What are the most important things that [the challenges of memory] interfere with*?*”* (Psychologist, interview 2)

#### Strengths regarding memory

Strengths or potential strengths for this polarity were explored with a focus on the power of sharing memories and being the shared memory. The more intense access to distant memories, such as those of childhood, was also found to be possible source of cheerfulness and delight in the group discussions run by the psychologist. A key facilitating question in this regard was:

*“What are cherished or pleasurable memories*?*”* (Psychologist, interview 1)

For the discontinuity of forgetting, professionals learned to make special note of important meanings, and needs, so that they could quote these back to their clients later on. In so doing they tried to help strengthen the memory function by sharing it. Two guidelines were experienced as important in this regard. One, sharing should not be an authoritative, top down process, but more an open offering, so as not to be confrontational with regard to forgetting:

*“I say things like*: *‘a while back we talked about so and so*, *you mentioned that these things were important*, *do you recognize that [as personally important]*?*’*” Case manager 1, interview 1)

Two, referring back to things that were important in the past helped strengthen especially if the professional could “frame it lovingly”, that is with an explicit invested concern:

*“What touched me last time we spoke about this subject is that [these things] seemed to really be important for you”* (Psychologist, interview 1)

For both poles, professionals also stressed the importance of involving loved ones in sharing cherished memories or framing memory lovingly. Some way to capture or anchoring these memories, for instance through photo books or diaries, was held to be an important way to continue to share memory lovingly beyond the early stage of dementia.

#### Believing–Knowing: exploring one’s predicament

With respect to the *believing—knowing* polarity, people struggle with ways to *surrender* to their situation or with finding something that they can always rely upon or be *certain* of. Herein issues of faith and meaning are often central. Case managers and psychologist all spoke of the prevalence of this polarity in the lives of their clients and their loved ones. It was the psychologist though, who in two discussion groups about coming to grips with the diagnosis and implications of dementia, made most room to fully explore these struggles. In those groups an important opening question was:

*“What do you face with regard to living with dementia*?*”* (Psychologist, interview 1)

#### Tensions regarding ones predicament

In the groups multiple clients told stories of the experienced fear surrounding trying to come to grips with the meaning of the diagnosis and the (possible) consequences of dementia on their lives. This fear kept them from giving themselves over to their current situation. In the discussion groups these sentiments were explored through these questions:

*“What images do you associate with dementia*?*”* (Psychologist, interview 1)

*“What are fears for the future*?*”* (Psychologist, interview 1)

For people in the peer groups and also in supportive conversations of case managers, tension could also be experienced concerning what to expect in moving forward. For them a search for certainty was more prevalent. In the further exploration, professionals often offered some explanatory framework about what is and what is not known about dementia, but often left the explanation open ended:

*“I try to help somewhat in this area by explaining that we can’t be sure about how the disease will manifest itself*. *That we know for certain that you won’t get better*, *but that the particular process of the disease is unique for each individual*.” (Psychologist, interview 1)

Case managers also reported that this search for certainty was especially an ongoing source of tension for loved ones. For them, mysterious changes in the way of being of the person with dementia often caused fundamental uncertainty about how to understand what was going on. Case managers, often then explored with the loved one what might be the dementia related causes of shifts in behavior:

*“Often we then try to interpret changes in their partner together*. *During that exploration I do offer a possible ‘disease perspective’*, *based on what we know to often go together with a certain type of dementia*.*”* (Case manager 1, interview 2)

A helpful question here was:

*“What could the influence of dementia be [on the experienced changes]*?*”* (Case manager 1, interview 2)

#### Key transformative questions

Key questions found to facilitate a core perspective on and (a bit more) inner space around the tensions was:

*“What is most important for quality of life*?*”* (Psychologist, interview 1)

*“What do we need to know to be able to deal with the situation*?*”* (Case manager 1, interview 2)

#### Strengths regarding ones predicament

With respect to strengths, explorations case managers, especially after noticing spiritual or religious symbols around the house or on the person, would explore the significance and possible power of a person’s spiritual beliefs, traditions and rituals with questions such as:

*“What are your spiritual beliefs*?*”* (Case manager 5, interview 1)

*“How do they help you deal with dementia/ the current challenges*?*”* (Case manager 5, interview 1)

The psychologist in her peer groups also explored special activities or pastimes that people could surrender to with the following questions:

*“Are there special activities or pastimes that you can totally loose yourself in*? *That you are deeply at peace or one with*?*”* (Psychologist, interview 1)

Answers to these questions could also lead to further exploration of explicit spiritual strengths such as prayer, but also to less implicit ones such as being at one with nature, music or art. With respect to potential strengths found in what you can rely upon, the psychologist gave much room to explore a possible strength giving overview of ones life with the questions:

*“What are you grateful for in life*?*”* (Psychologist, interview 1)

*“Where do you feel blessed*?*”* (Psychologist, interview 2)

Through such explorations people were regularly found to experience a more positive meaning of their life story as a whole, which gave them something to hold onto. These explorations could extend to all other strength related aspects of the other polarities in the model, informing an overarching story of “the beautiful life”.

## Discussion

The main goal of our research was to find and develop ways of exploring existential and spiritual issues that help enhance an experience of inner space for people with mild to moderate dementia and their loved ones. In our participatory action research with dementia care professionals we have further developed the Diamond model for conversation support to include these types of exploration.

### Main findings

Regarding the enhancement of inner space through conversations, a few general insights were found to be important for professionals. First, this kind of support starts with being able to *recognize* when existential or spiritual issues are prevalent within the conversation. Cues for recognizing such issues were seen as the experience of tension within the client and or loved one, or a need to grow and bolster strengths. Second, supporting people with regard to tensions and strengths ideally required the professional to make space in themselves, to explore issues in an open and receptive manner. This often required them to suspend their own conversational agenda in favor of a *basic attitude of a co-researcher*. Third, the *exploration of tensions is often leading* in necessity. Not only because clients and loved ones often have a strong need to alleviate that tension, but also because they are less receptive to the exploration of (possible) strengths if tension is dominant. Fourth, following thorough exploration of tensions, the client will often experience a moment of *pregnant silence*. *Key transformative questions* were found to be especially helpful for the enhancement of inner space following this silence. Answers to these questions were also found to open up room in the conversation to explore specific existential or spiritual strengths. Exploratory approaches found to be helpful for the enhancement of inner space often followed this *conversational sequence*.

**Fig 2:**
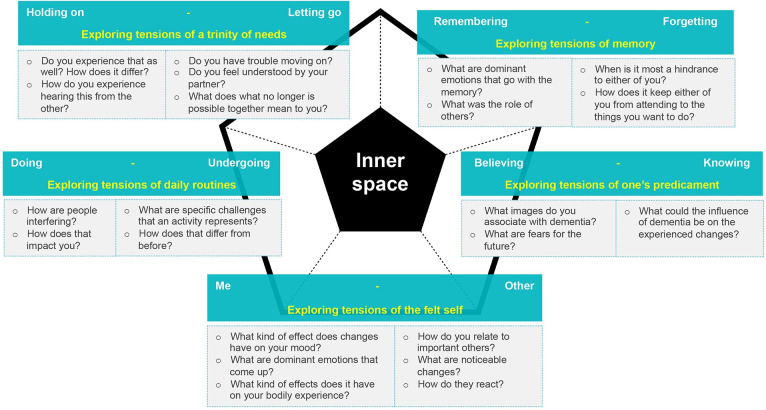
The Diamond tension questions for mild to moderate dementia.

**Fig 3:**
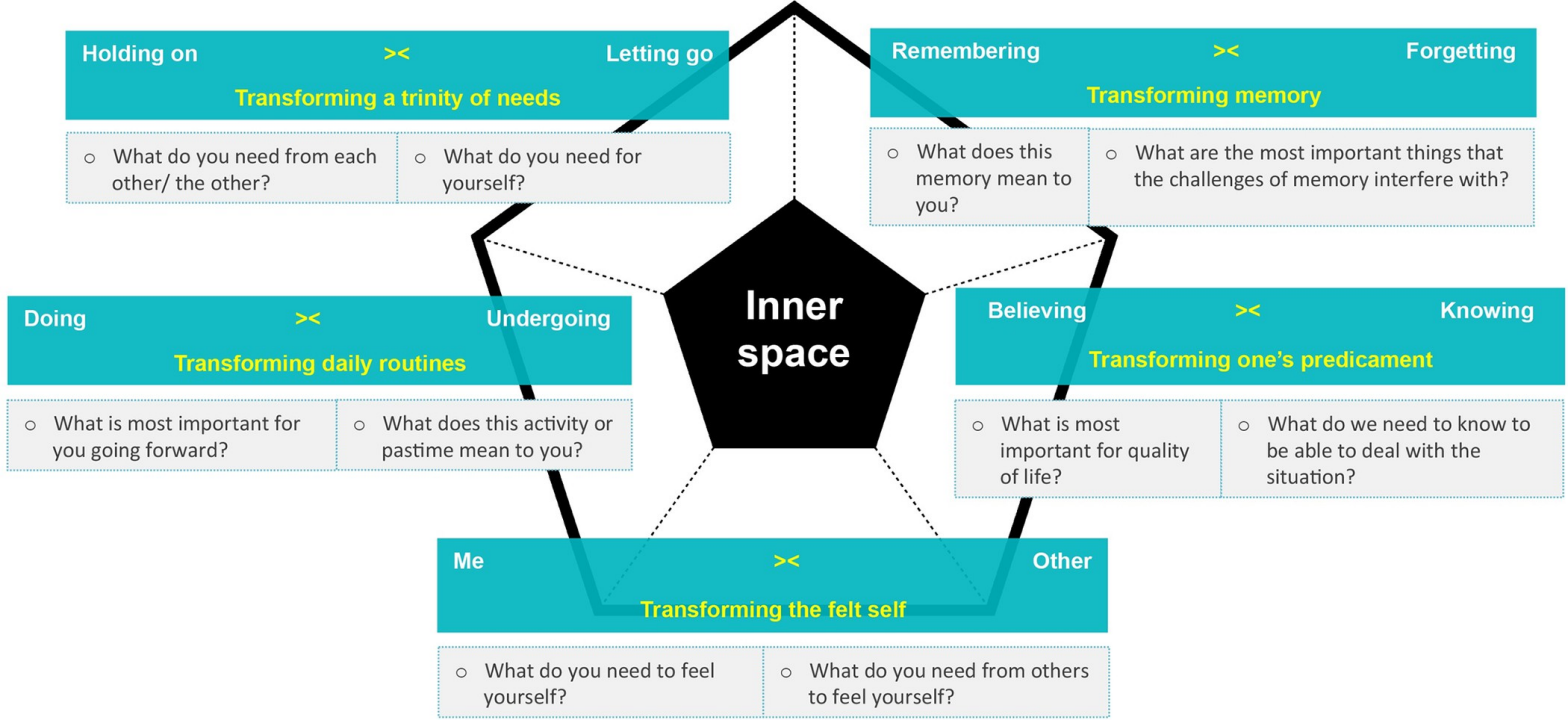
The Diamond transformative questions for mild to moderate dementia.

**Fig 4:**
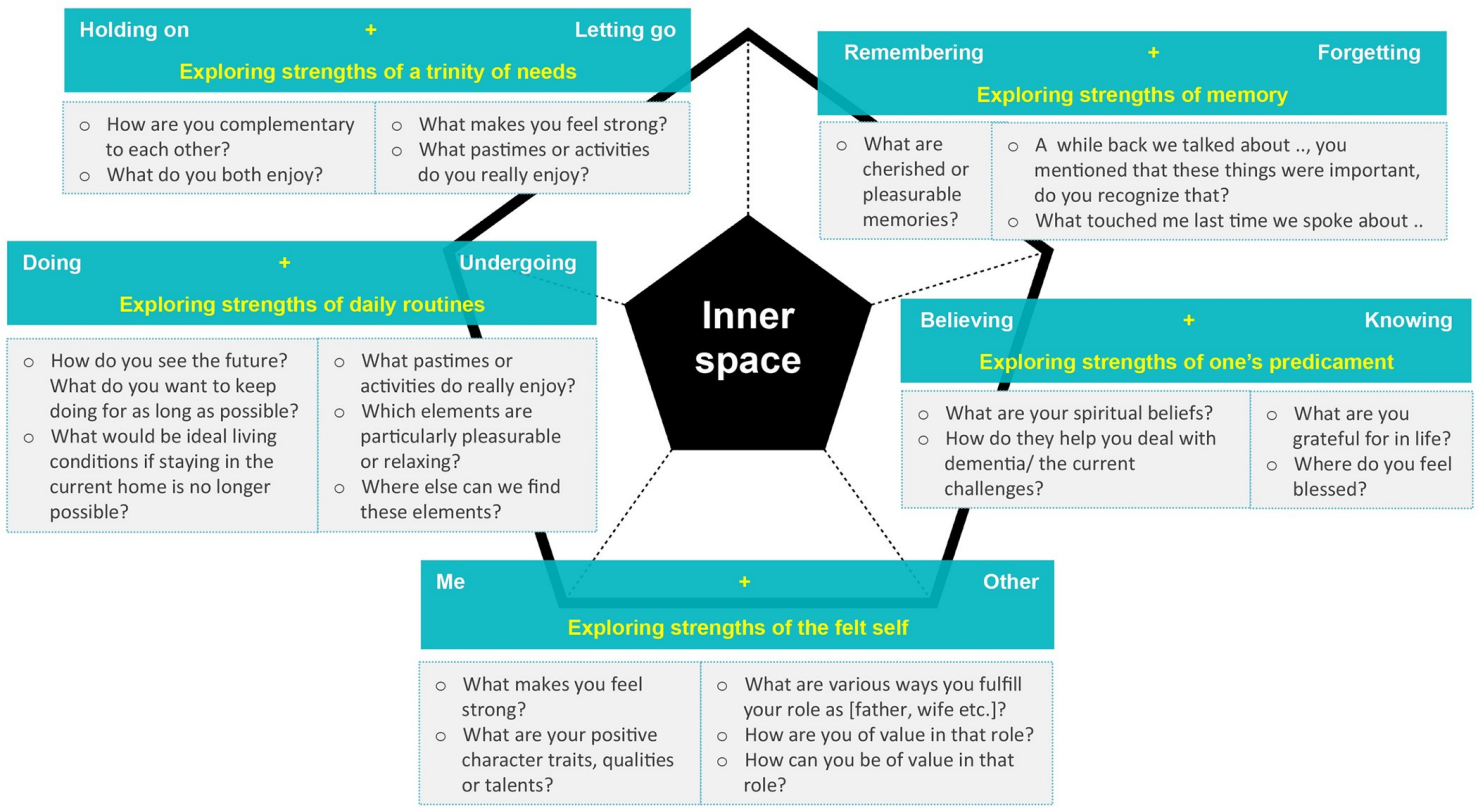
The Diamond strength questions for mild to moderate dementia.

The framework of the Diamond model for early stage dementia helped unearth, develop and order these approaches in: *exploring the felt self* for the me-other polarity, *exploring daily routines* for the doing-undergoing polarity, *exploring a trinity of needs* for the holding on- letting go polarity, *exploring memory* for the remembering-forgetting polarity and *exploring one’s predicament* for the believing-knowing polarity. Our participatory action research yielded a variety of exploratory questions and prompts that facilitated exploration. Figs 2–4 show the questions and prompts per approach for the sequence of tensions, transformation and strengths respectively.

This sequence in conversational support, within, or a cross conversations, was found to be the clearest pathway to transformation. However, coming to grips with and giving focused voice to what a tension is, was also found to be existentially and spiritually rewarding in its own right. For some, just getting to the heart of the tension is enough to make space and set free. For many, exploring strengths thereafter, could further help recognize or create signposts of transformation and positive reinforcement in the existential or spiritual dimension. For others, an opportunity to grow and live more from strength was leading. For them, exploring tensions was not primary. In this sense the found sequence should not be taken as prescriptive for all conversational support. Rather, the components of the approaches can be seen as types of exploration that can be applied according to what best fits with the needs of the person with dementia and their loved ones in the moment.

#### Relation to previous research, strengths and limitations

As mentioned in the introduction, there is a lack of existential or spiritual conversation tools that can aid dementia care professionals in their support of clients and loved ones. This is especially so for people with mild to moderate dementia [11]. Literature concerning *conversational* support mostly focuses on contextual (such as a quiet surrounding), non-verbal (for instance using touch), para-lingual (for instance speaking slowly) or semantic (such as avoid abstract language) conditions [12, 26]. Literature regarding *spiritual* support for people with mild to moderate dementia, strongly tends to focus on spirituality as religious background and how to better connect with that tradition or community [2, 10]. Such a view of the spiritual may lead non-religious professionals to disengage with this type of support and defer to the church [7]. To cover a broader spectrum and enhance experienced responsibility, new understandings and tools are needed. Based on our research we support Ødbehr’s [9] definition of what existential and spiritual support can be, following research on the views of clients and caregivers themselves: *‘‘Meeting people where they are and assisting them in connecting or re-connecting to things*, *practices*, *ideas and principles that are their core of their being–the breath of their lives”*.

As far as we know, the developed and tested Diamond model is the first framework to aid professionals in providing ongoing existential and spiritual support to reconnect people with mild to moderate dementia and their loved ones to the core of their being. In her evaluation of spiritual care in a dementia care setting, Goodal [7] did come to a recommendation of three steps from the view of the professional: one, reflect upon on the person and situation to asses what is needed, two, realize what is needed through a co-constructive relationship and three, asses whether the relationship leads to an experience of restoration for the resident. Our model goes much further by making explicit how a co-constructive relationship may work in conversation. It also makes the assessment of what is needed *in itself* a co-constructive activity, through a relationship of co-researching. Lastly, we re-conceptualize and differentiate the purported goal of this type of support to go beyond the narrower notion of restoration. The enhancement of inner space, as an experience of release from inner tension, a freedom to relate to pressing concerns in a new way, and an opening up of (trans) formative possibility, allows for reconnection with what is of deep importance. The purpose is therefore to restore this deep connection, in ways that allow for new and enlightening modes of being. Hereby the found (components of) approaches can help structure conversational support in ways that best befit the specific needs and situation of clients at that moment. Two questions with regard to exploring strengths of daily routines also address possible future needs and living situations. These questions were successful in enhancing a sense of direction and control regarding the future. However, there is evidence to suggest that people with mild dementia can resist conversations about future arrangements because they do not want to be confronted with a future diminishment in autonomy [27]. Based on our research we can say that the timing for questions about future arrangements does seem most promising when clients are already experiencing challenges, such as getting up and down stairs, that press upon a sense of autonomy. Explorations of wishes for the future can then also have empowering effects in the now, as clients become involved in making supportive choices for their foreseeable future selves.

Within the literature we have found that certain forms of spiritual reminiscence offer some questions that are similar to those in the Diamond framework. MacKinlay and Trevitt [28] for instance, formulate good reminiscence as “*asking people questions about*: *what gives their life meaning; whether they perceive that life is worth living; how they feel about getting older; what they find most difficult in life; their hopes*, *joys*, *fears and regrets; the spiritual and/or religious practices that are important to them; and their relationship with God or a higher power”*. Our research confirms that some of these types of questions are helpful in conversational support. According to the reminiscence approach, the professional sets the course of what to explore. The Diamond framework, however, does not act as an agenda for an interview. It allows professionals to meet people where they are in terms of tensions and strengths, related to five existential or spiritual polarities, which have been found to be of fundamental importance. It also offers insight into the possible flow of supportive conversations, with an eye toward, but not a necessity of, transformation. It gives the professional more insight into the possible process and dynamics of different types of conversations. This makes the framework at the same time more flexible and more specific in its facilitation of support than reminiscence.

Of course our research and the resulting framework has its limitations as well. Although the model has been developed and tested through conversations with 62 clients and 32 loved ones, only six professionals participated in our study. It is probable that the participation of more professionals would have yielded a somewhat different span of questions and prompts.

Participating professionals did not receive additional training in existential and spiritual communication before trying out and further developing the dementia-Diamond model framework. The reason for this is that there were no specific Diamond model conversational approaches available at the beginning of our research and we wanted to develop these explicitly within the Diamond model framework through trial and error learning, so as to ground them in practice as much as possible.

Also, participants’ professional role and conversational setting has undoubtedly impacted the way in which they further developed the Diamond model in their practice. Though all Diamond polarities were encountered regularly in the conversations, there was a difference in what felt natural for professionals to give more space. The peer groups run by the psychologist for instance, offered more space to delve deeper intra-personally concerning tensions and strengths of the felt self and one’s life predicament. Case managers, often more practical trouble shooters in their conversations with clients *and* their loved ones, had a more natural inclination to delve deeper into daily routines and a trinity of needs. It is therefore quite possible that other types of professionals, such as chaplains, social workers or counselors would have developed different routes and questions more in tune with their work setting and professional background.

Lastly, with regard to methods, the conducted interviews where coded by the principle researcher and not by two researchers independently. It is possible that the coding of fragments would have been somewhat different had the latter occurred. Since participants always explicitly noted when conversational approaches and questions led to more inner space with clients or had promise to do so, we feel confident that possible coding bias was kept to a minimum. The found approaches were furthermore member checked at the end of the research process.

### Scope of application

So how can the Diamond model for dementia best be seen in light of these limitations? In our view the current framework can be applied in four ways. One, the differentiation of existential and spiritual issues and approaches within the polarities can help professionals navigate the conversational flow and help them generate their own questions in the mutual exploration of issues. Two, the framework can be used by professionals to reflect on their ongoing conversations, to check whether they are on the right track, or whether they are missing a potentially important angle for exploration. Three, the polarity questions can be memorized, so that professionals have at their disposal sets of questions that they can use if the conversation organically warrants them. Fourth, and finally, the framework can be used in team or multidisciplinary discussions to gain a clearer understanding of what existential or spiritual support could entail beyond the scope of the strictly religious. In this way the framework can help to create a shared language and act as a sounding board for the development and improvement of organizational support. All these application forms can help professionals widen their understanding of what is possible and needed with regard to existential or spiritual conversations.

With regard to training dementia care professional to apply the framework and found approaches, we have found participatory action research principles to be very beneficial. By relating the Diamond model for dementia to ones own practice, trying out ways of support inspired by the framework and reflecting together on what can lead to more inner space in specific situations, professionals not only make the framework their own, but also learn to apply it creatively. Sharing these experience with one another has the added advantages of learning from each other and creating a shared language for this type of support. In order to overcome the obstacle of trying out something new in an already busy schedule though, management and team leadership needs to be committed to developing spiritual care and create opportunities and incentive to do so. This latter can be accomplished for instance by using already scheduled team or case study meetings for shared reflection on the application process.

### Further research and conclusion

Our research represents a starting point that can be further developed and tested in practice with more professionals, a greater variety of professionals and settings and with populations in other countries. The Diamond model for mild to moderate dementia offers a tested set of approaches, connected to core issues and polarities, within an overall flow of working towards the enhancement of a sense of newfound meaning, freedom and possibility. Extending this type of work and research is not only valuable, but, in light of the sheer scale of dementia in our societies, a necessity.
